# Annexin A5-DM1 protein-drug conjugate for the treatment of triple-negative breast cancer

**DOI:** 10.1186/s43556-023-00167-7

**Published:** 2024-02-19

**Authors:** Alexis Woodward, Benjamin Southard, Sampurna Chakraborty, Aaron O. Bailey, Gabriela N. F. Faria, Patrick McKernan, Wajeeha Razaq, Roger G. Harrison

**Affiliations:** 1 Stephenson School of Biomedical Engineering, University of Oklahoma, Norman, OK USA; 2 Department of Biochemistry and Molecular Biology, University of Texas Medical Branch, Galveston, TX USA; 3 AbCellera Biologics Inc., Vancouver, BC Canada; 4 School of Sustainable Chemical, Biological, and Materials Engineering, University of Oklahoma, Norman, OK USA; 5 Stephenson Cancer Center, Oklahoma City, OK USA

**Keywords:** Annexin A5, DM1, Breast cancer, Immunogenic cell death, Protein drug conjugate

Triple-negative breast cancer (TNBC) is defined by the absence of estrogen, progesterone, and human epidermal growth factor 2 hormonal receptors rendering the triple-negative phenotype. TNBC is the most aggressive and metastatic type of breast cancer and has the lowest 5-year survival rate of all breast cancers. Despite many efforts to develop a targeted treatment, TNBC has been coined an orphan disease, and the standard of care for TNBC patients remains untargeted conventional chemotherapy, radiation, and/or surgery. However, the aminophospholipid phosphatidylserine (PS) provides an innate biomarker on TNBC cells for the targeted treatment of this disease.

In the mammalian plasma membrane, PS is the only anionic phospholipid and comprises up to 10% of the total lipid content [1]. Despite its abundance, the asymmetry of PS across the plasma membrane is tightly regulated and plays a confounding role in regulating cell death and removal by the innate immune system. In healthy cells, PS exclusively resides within the cytosolic leaflet of the plasma membrane, but as cells become stressed/apoptotic, PS is flipped to the extracellular leaflet where it promotes the removal of the sick cells without activating the adaptive immune system [2]. During neoplastic transformation, the asymmetry of PS across the plasma membrane is also lost, leading to increased exposure of PS on the extracellular leaflet. Cancerous cells have up to seven times more external PS than healthy cells, and PS is highly externalized in metastatic TNBC [1]. However, this increase in PS externalization leads to an immunosuppressive microenvironment, and the cancerous cell survives [1, 2]. Despite its role in cancer progression, PS creates a unique biomarker for targeted chemotherapies.

Proteins of the annexin family are natural binding proteins to PS. Annexins are a diverse family of proteins that play a critical role in the homeostasis of cellular membranes. These small proteins are characterized by their ability to bind to negatively-charged phospholipids in a Ca^2+^-dependent manner. The most notable among annexin family members is annexin A5 (ANXA5), which is a main component in many commercial kits used to detect apoptosis. ANXA5 has a high specificity and affinity for externalized PS on TNBC cells as indicated by the low dissociation constant in the low nanomolar range, while no ANXA5-PS binding was observed on healthy MCF10A breast cancer cells **(**Supplemental Table 1 and Supplemental Fig. 2). After binding, ANXA5 enters the cell through a non-receptor-mediated pathway. ANXA5 binds to PS, and the protein crystallizes into a trimer network, bending the plasma membrane inward and forces internalization [3]. Our group and others have employed the binding of ANXA5 to PS to target antineoplastic agents to the tumor surface [1].

Here, we present a unique therapeutic strategy targeting the expression of PS in TNBC with its natural binding protein, ANXA5. By covalently linking ANXA5 and emtansine (DM1) together through sulfo-SMCC crosslinking, we create a novel bioconjugate (ANXA5-DM1) for the targeted treatment of TNBC (Fig. 1a). DM1 is a potent anticancer drug that inhibits microtubule formation and induces immunogenic death cell (ICD) both in vitro and in vivo when linked to an antibody [4, 5]. The ANXA5-DM1 conjugate displayed TNBC-specific and enhanced cytotoxicity when compared to free DM1, and importantly, the ANXA5-DM1 conjugate did not have cytotoxic effects against healthy breast cells. Finally, we show the ANXA5-DM1 conjugate increased the release or externalization of two hallmark ICD damage-associated molecular patterns (DAMPs).

**Fig. 1:**
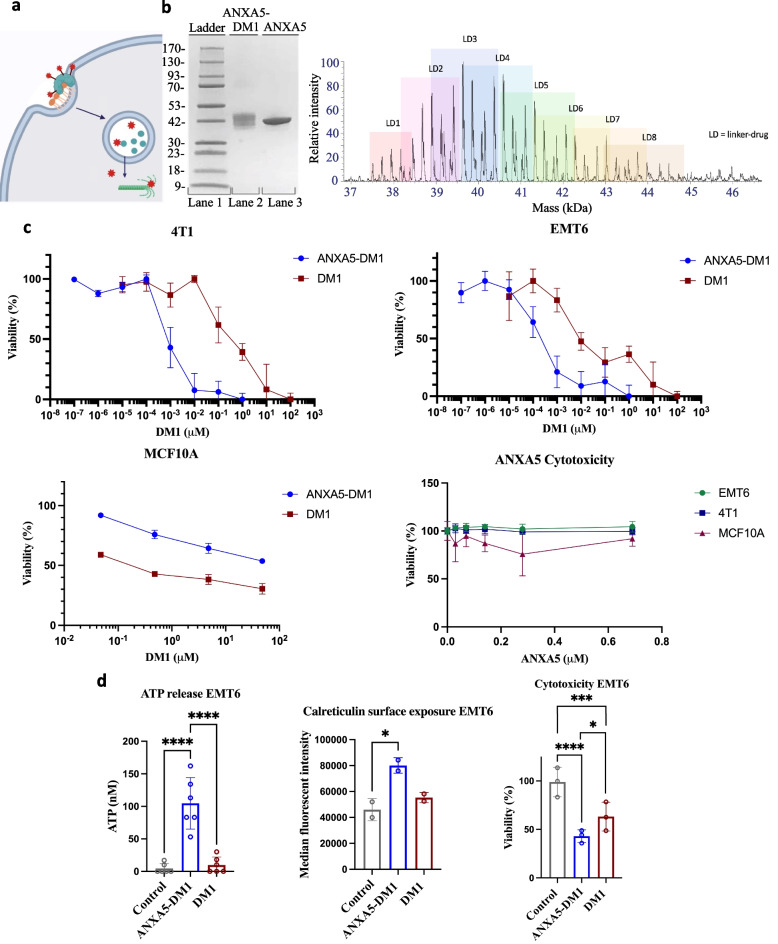
Mechanism, characterization, and anticancer effects of the ANXA5-DM1 conjugate on triple-negative breast cancer. **a** ANXA5-DM1 (teal protein linked to red stars) binds to PS-expressing (orange phospholipid) cancer cells. ANXA5-DM1 is internalized and ANXA5 (teal protein) is broken down in the lysosome. Free DM1 (red stars) diffuses out of the lysosome and causes mitotic catastrophe. Image created with Biorender.com. **b** Left: SDS-PAGE of the ANXA5-DM1 bioconjugate (lane 2) compared to free ANXA5 (lane 3). The increase in molecular weight of the conjugate compared to ANXA5 is estimated to be 6 ± 3 kDa. Right: Denaturing intact mass spectrometry analysis. Each linker-drug adds 957 Da indicated by the pastel boxes. The ANXA5-DM1 conjugate had a range of 1 to 8 linker(s) and drug(s) added. The weighted average of ANXA5-DM1 was 3.9 molecules of DM1 to 1 molecule of ANXA5. **c** 72-h cytotoxic effects of ANXA5-DM1, free DM1, and free ANXA5 on mouse 4T1 triple-negative breast cancer cells, mouse EMT6 triple-negative breast cancer cells, and 100% confluent healthy MCF10 mammary cells. The concentration of DM1 as the free drug (red squares) or in the ANXA5-DM1 conjugate (blue circles) is shown. Top left: 4T1 IC50 values: 0.85 nM for ANXA5-DM1 and 320 nM for free DM1 (data presented as mean ± SD with *n* = 4). Top right: EMT6 IC50 values: 0.21 nM for ANXA5-DM1 and 28 nM for free DM1 (data presented as mean ± SD with *n* = 4). Bottom left: MCF10A IC50 values: 160 nM for free DM1, and > 5000 nM for ANXA5-DM1 (data presented as mean ± SD with *n* = 3). Bottom right: There was no statistically significant change in cell viability as the ANXA5 concentration increased for 4T1, EMT6, and MCF10 cells (data presented as mean ± SD with *n* = 5 for 4T1 cells and with *n* = 3 for EMT6 and MCF10 cells). **d** Immunogenic cell death (ICD) activity of 10 nM ANXA5-DM1 and DM1 on EMT6 cells. ICD activity was measured through ATP release, calreticulin surface expression, and cytotoxicity after 24 h. Left: For ATP release, there was a significant increase for ANXA5-DM1 treatment compared to free DM1 treatment or the control (data represented as mean ± SD,  *n* = 6). Center: For calreticulin surface expression, there was a significant increase for ANXA5-DM1 treatment compared to free DM1 treatment or the control (data represented as mean ± SD, *n* = 2 with each sample run in triplicate). Right: For cytotoxicity activity, the cell death of EMT6 cells increased significantly for ANXA5-DM1 treatment compared to free DM1 treatment or the control (data represented as mean ± SD, *n* = 3). Statistical significance is denoted by *(*p* < 0.0332), **(*p* < 0.0021), ***(*p* < 0.0002), and **** (*p* < 0.0001)

ANXA5-DM1 was successfully conjugated together through sulfosuccinimidyl 4-(N-maleimidomethyl)cyclohexane-1-carboxylate (sulfo-SMCC) (see Supplementary Information for details). ANXA5-DM1 was characterized through absorbance at 288 nm (Supplemental Fig. 1), SDS-PAGE, and denaturing intact mass analysis. SDS-PAGE showed a molecular weight increase of 6 ± 3 molecules following DM1 (MW 740 Da; MW 960 Da with crosslinker) addition (Fig. 1b, left). By deconvoluting the ANXA5-DM1 conjugate in the mass range of 35–45 kDa of denaturing intact mass analysis, a left-shifted bell curve distribution of ANXA5-DM1 is observed (Fig. 1b, right). The weighted average of the drug-to-protein ratio obtained was 3.9 molecules of DM1 to 1 molecule of ANXA5, which is within the range previously found with SDS-PAGE analysis.

Next, we examined the cytotoxicity using a 72 h in vitro assay of the ANXA5-DM1 conjugate and free DM1 in mouse 4T1 and EMT6 triple-negative breast cancer cells and in MCF10A healthy mammary cells. For TNBC cells, the antineoplastic activity of DM1 was significantly enhanced as part of an ANXA5 bioconjugate (Fig. 1c, top). Measuring the inhibitory concentration of 50 percent (IC50) in a 72 h in vitro viability assay, the ANXA5-DM1 bioconjugate was more than 2 orders of magnitude more potent than free DM1 in the EMT6 and 4T1 cell lines. The ANXA5-DM1 conjugate was significantly less cytotoxic to healthy MCF10A cells grown to 100% confluence than the free DM1 at doses up to 48 μM (Fig. 1c, bottom left). The IC50 of the ANXA5-DM1 could not be determined and exceeded 48 μM; however, the IC50 for free DM1 was 160 nM. Compared to the two TNBC cell lines studied, the 48 μM dose of DM1 in the ANXA5-DM1 conjugate is 56,000 to 229,000 times larger than the IC50 for the noncancerous mammary cells. This indicates the ANXA5-DM1 conjugate is considerably less toxic to healthy breast cells than it is to cancer cells.

To ensure DM1 is causing the cytotoxic action of the conjugate, cytotoxicity studies of ANXA5 were carried out on TNBC cells as well as healthy mammary cells. The presence of ANXA5 on two TNBC cell lines and one healthy mammary cell line did not significantly impact viability with doses up to 0.7 μM (Fig. 1c, bottom right). At the higher IC50 of ANXA5-DM1 for the TNBC cells (0.85 nM DM1), the equivalent IC50 based on the ANXA5 concentration is 0.21 nM, assuming 4 mol DM1 per mole ANXA5; this concentration is 3290 times less than the highest ANXA5 concentration tested for free ANXA5 (690 nM, Fig. 1c, bottom right). This indicates that the cytotoxic action of the conjugate is a result of the addition of the drug and not the protein alone.

Finally, we examined ANXA5-DM1's ability to induce ICD by evaluating the release of ATP into the extracellular space and calreticulin surface expression. At 10 nM, ANXA5-DM1 significantly increased ATP release (Fig. 1d, left). ANXA5-DM1 caused nearly 10 times more ATP released than free DM1. Cancer cells require large stores of ATP to undergo constant division, protein production, and cell signaling, so, unsurprisingly, the untreated control had essentially 0 ATP in the extracellular space. Incubation with ANXA5-DM1 significantly increased calreticulin surface expression in comparison to the untreated control as determined by median fluorescent intensity (Fig. 1d, center). Although both ANXA5-DM1 and free DM1 significantly increased cell death (Fig. 1d, right), only ANXA5-DM1 significantly increased ICD DAMPs. Additionally, ANXA5-DM1 significantly increased cell death in comparison to free DM1.

In summary, we have successfully conjugated DM1 to ANXA5 for the targeted treatment of TNBC. We find that the ANXA5-DM1 conjugate quickly initiates cell death and induces two hallmarks of ICD.

### Supplementary Information

**Additional file 1.**

## Data Availability

All data generated or analyzed during this study are included in this published article.
