# Correction: annexin A5-DM1 protein-drug conjugate for the treatment of triple-negative breast cancer

**DOI:** 10.1186/s43556-024-00180-4

**Published:** 2024-04-02

**Authors:** Alexis Woodward, Benjamin Southard, Sampurna Chakraborty, Aaron O. Bailey, Gabriela N. F. Faria, Patrick McKernan, Wajeeha Razaq, Roger G. Harrison

**Affiliations:** 1 Stephenson School of Biomedical Engineering, University of Oklahoma, Norman, OK USA; 2 Department of Biochemistry and Molecular Biology, University of Texas Medical Branch, Galveston, TX USA; 3 AbCellera Biologics Inc, Vancouver, BC Canada; 4 School of Sustainable Chemical, Biological, and Materials Engineering, University of Oklahoma, Norman, OK USA; 5 Stephenson Cancer Center, Oklahoma City, OK USA

**Correction: Mol Biomed 5, 7 (2024)**

**https://doi.org/10.1186/s43556-023-00167-7**

Following publication of the original article [1], the authors reported that Fig. 1 needed to be updated because the log axis for the DM1 concentration is incorrect in Fig. 1c. This error was caused by using an incorrect log axis when this figure was converted from black and white to color. The correct figure and caption are given hereafter.

The incorrect Fig. 1:

**Fig. 1:**
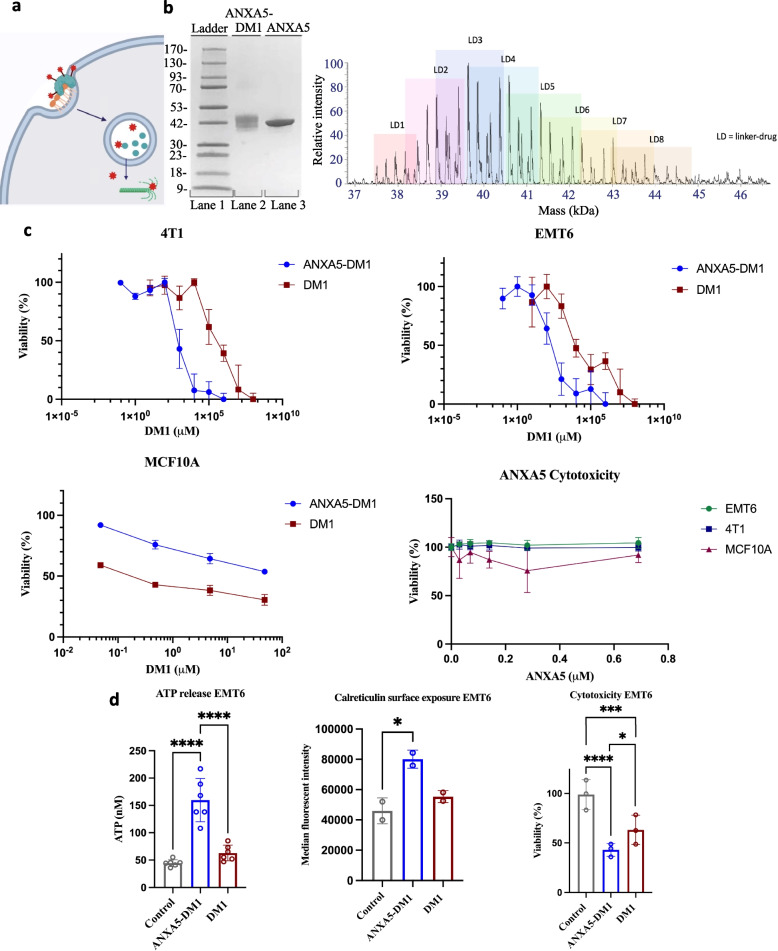
Mechanism, characterization, and anticancer effects of the ANXA5-DM1 conjugate on triple-negative breast cancer. **a** ANXA5-DM1 (teal protein linked to red stars) binds to PS-expressing (orange phospholipid) cancer cells. ANXA5-DM1 is internalized and ANXA5 (teal protein) is broken down in the lysosome. Free DM1 (red stars) diffuses out of the lysosome and causes mitotic catastrophe. Image created with Biorender.com. **b** Left: SDS-PAGE of the ANXA5-DM1 bioconjugate (lane 2) compared to free ANXA5 (lane 3). The increase in molecular weight of the conjugate compared to ANXA5 is estimated to be 6 ± 3 kDa. Right: Denaturing intact mass spectrometry analysis. Each linker-drug adds 957 Da indicated by the pastel boxes. The ANXA5-DM1 conjugate had a range of 1 to 8 linker(s) and drug(s) added. The weighted average of ANXA5-DM1 was 3.9 molecules of DM1 to 1 molecule of ANXA5. **c** 72-h cytotoxic effects of ANXA5-DM1, free DM1, and free ANXA5 on mouse 4T1 triple-negative breast cancer cells, mouse EMT6 triple-negative breast cancer cells, and 100% confluent healthy MCF10 mammary cells. The concentration of DM1 as the free drug (red squares) or in the ANXA5-DM1 conjugate (blue circles) is shown. Top left: 4T1 IC50 values: 0.85 nM for ANXA5-DM1 and 320 nM for free DM1 (data presented as mean ± SD with *n* = 4). Top right: EMT6 IC50 values: 0.21 nM for ANXA5-DM1 and 28 nM for free DM1 (data presented as mean ± SD with *n* = 4). Bottom left: MCF10A IC50 values: 160 nM for free DM1, and > 5000 nM for ANXA5-DM1 (data presented as mean ± SD with *n* = 3). Bottom right: There was no statistically significant change in cell viability as the ANXA5 concentration increased for 4T1, EMT6, and MCF10 cells (data presented as mean ± SD with *n* = 5 for 4T1 cells and with *n* = 3 for EMT6 and MCF10 cells). **d** Immunogenic cell death (ICD) activity of 10 nM ANXA5-DM1 and DM1 on EMT6 cells. ICD activity was measured through ATP release, calreticulin surface expression, and cytotoxicity after 24 h. Left: For ATP release, there was a significant increase for ANXA5-DM1 treatment compared to free DM1 treatment or the control (data represented as mean ± SD, *n* = 6). Center: For calreticulin surface expression, there was a significant increase for ANXA5-DM1 treatment compared to free DM1 treatment or the control (data represented as mean ± SD, *n* = 2 with each sample run in triplicate). Right: For cytotoxicity activity, the cell death of EMT6 cells increased significantly for ANXA5-DM1 treatment compared to free DM1 treatment or the control (data represented as mean ± SD, *n* = 3). Statistical significance is denoted by *(*p* < 0.0332), **(*p* < 0.0021), ***(*p* < 0.0002), and **** (*p* < 0.0001)

The correct Fig. 1:

**Fig. 1:**
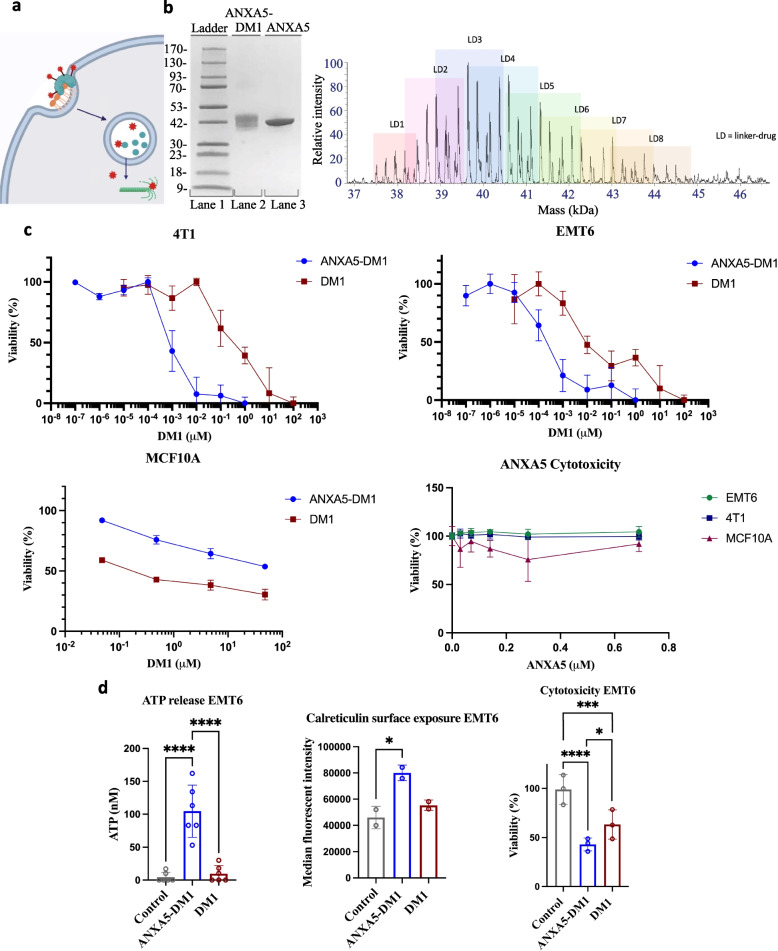
Mechanism, characterization, and anticancer effects of the ANXA5-DM1 conjugate on triple-negative breast cancer. **a** ANXA5-DM1 (teal protein linked to red stars) binds to PS-expressing (orange phospholipid) cancer cells. ANXA5-DM1 is internalized and ANXA5 (teal protein) is broken down in the lysosome. Free DM1 (red stars) diffuses out of the lysosome and causes mitotic catastrophe. Image created with Biorender.com. **b** Left: SDS-PAGE of the ANXA5-DM1 bioconjugate (lane 2) compared to free ANXA5 (lane 3). The increase in molecular weight of the conjugate compared to ANXA5 is estimated to be 6 ± 3 kDa. Right: Denaturing intact mass spectrometry analysis. Each linker-drug adds 957 Da indicated by the pastel boxes. The ANXA5-DM1 conjugate had a range of 1 to 8 linker(s) and drug(s) added. The weighted average of ANXA5-DM1 was 3.9 molecules of DM1 to 1 molecule of ANXA5. **c** 72-h cytotoxic effects of ANXA5-DM1, free DM1, and free ANXA5 on mouse 4T1 triple-negative breast cancer cells, mouse EMT6 triple-negative breast cancer cells, and 100% confluent healthy MCF10 mammary cells. The concentration of DM1 as the free drug (red squares) or in the ANXA5-DM1 conjugate (blue circles) is shown. Top left: 4T1 IC50 values: 0.85 nM for ANXA5-DM1 and 320 nM for free DM1 (data presented as mean ± SD with *n* = 4). Top right: EMT6 IC50 values: 0.21 nM for ANXA5-DM1 and 28 nM for free DM1 (data presented as mean ± SD with *n* = 4). Bottom left: MCF10A IC50 values: 160 nM for free DM1, and > 5000 nM for ANXA5-DM1 (data presented as mean ± SD with *n* = 3). Bottom right: There was no statistically significant change in cell viability as the ANXA5 concentration increased for 4T1, EMT6, and MCF10 cells (data presented as mean ± SD with *n* = 5 for 4T1 cells and with *n* = 3 for EMT6 and MCF10 cells). **d** Immunogenic cell death (ICD) activity of 10 nM ANXA5-DM1 and DM1 on EMT6 cells. ICD activity was measured through ATP release, calreticulin surface expression, and cytotoxicity after 24 h. Left: For ATP release, there was a significant increase for ANXA5-DM1 treatment compared to free DM1 treatment or the control (data represented as mean ± SD, *n* = 6). Center: For calreticulin surface expression, there was a significant increase for ANXA5-DM1 treatment compared to free DM1 treatment or the control (data represented as mean ± SD, *n* = 2 with each sample run in triplicate). Right: For cytotoxicity activity, the cell death of EMT6 cells increased significantly for ANXA5-DM1 treatment compared to free DM1 treatment or the control (data represented as mean ± SD, *n* = 3). Statistical significance is denoted by *(*p* < 0.0332), **(*p* < 0.0021), ***(*p* < 0.0002), and **** (*p* < 0.0001)

The original article [1] has been corrected.
