# Supplementary material for: Annexin A5-DM1 protein-drug conjugate for the treatment of triple-negative breast cancer
Source: Mol Biomed. 2024 Feb 19;5:7. doi: 10.1186/s43556-023-00167-7 (PMC10874913; doi:10.1186/s43556-023-00167-7)
Supplement: Supplementary file 1 — Additional file 1. [file 43556_2023_167_MOESM1_ESM.docx]

**Supplementary Information**

**Annexin A5-DM1 protein-drug conjugate for the treatment of triple-negative breast cancer**

Alexis Woodward,^1*^ Benjamin Southard,^1*^ Sampurna Chakraborty,^1^ Aaron O. Bailey,^2,3^ Gabriela N.F. Faria,^4^ Patrick McKernan,^1^ Wajeeha Razaq,^5^ and Roger G. Harrison,^4,5**^

^1^ Stephenson School of Biomedical Engineering, University of Oklahoma, Norman, OK, USA

^2^ Department of Biochemistry and Molecular Biology, University of Texas Medical Branch, Galveston, TX, USA

^3^ AbCellera Biologics Inc., Vancouver, BC, Canada

^4^School of Chemical, Biological and Materials Engineering, University of Oklahoma, Norman, OK, USA

^5^ Stephenson Cancer Center, Oklahoma City, OK, USA

**Methods**

Protein production

Recombinant ANXA5 was produced as previously described [1]. Briefly, recombinant ANXA5 was produced in BL21(DE3) *E. coli* transfected with a pET-30 Ek/LIC/ANXA5 plasmid. Bacteria were grown in Luria broth medium, and protein production was induced by isopropyl β-D-thiogalactopyranoside. The resulting protein was purified using an N-terminal polyhistidine-tag for purification by immobilized metal affinity chromatography (IMAC) with immobilized Ni^2+^ (GE Healthcare Life Sciences, Meadowvale, ON, Canada). An engineered HRV 3C protease cleavage site (LEVLFQ↓GP ) between the polyhistidine tag and ANXA5 enabled this tag to be removed by HRV 3C protease (Thermo Fisher Scientific, Waltham, MA, USA). The ANXA5 gene sequence was verified by DNA sequencing at the Oklahoma Medical Research Foundation (Oklahoma City, OK). Recombinant protein was confirmed as greater than 95% purity by SDS-PAGE and endotoxin free by Limulus assay (Thermo Fisher Scientific, Waltham, MA, USA).

Synthesis of annexin A5–emtansine (ANXA5-DM1) Conjugate

Mertansine (DM1- MedChemExpress, Monmouth Junction, NY, USA)) was linked to the primary amines (lysine residues) ANXA5 with the heterobifunctional crosslinker sulfosuccinimidyl 4-(N-maleimidomethyl)cyclohexane-1-carboxylate (sulfo-SMCC, TCI, Portland, OR, USA). First, 13.8 mM (6 mg/ml) of sulfo-SMCC is dissolved in DI water. If the sulfo-SMCC does not fully dissolve, the sulfo-SMCC mixture is gently heated to 40-50°C. The solution is allowed to cool to room temperature before the addition of the protein. Next, 200 μL of the sulfo-SMCC solution is added to 1 ml of 27.77 μM (1 mg/ml) ANXA5 and allowed to react for 1 h at 4°C on an orbital shaker. The maleimide moiety of the sulfo-SMCC linker readily captures the only thiol functional group in DM1. DM1 is dissolved in DMSO (Fisher Scientific, Pittsburgh, PA, USA) at a concentration of 9.03 mM (6.66 mg/ml). The DM1 mixture (150 μL) is added to ANXA5-sulfo-SMCC solution and allowed to react for 2 h at 4°C on an orbital shaker. The resulting ANXA5-DM1 biconjugate is then purified from excess free DM1 with 8-h dialysis in 2 L of 30 mM sodium phosphate buffer at pH 7.4 with a regenerated cellulose dialysis membrane of MWCO 12-14 kDa (Fisher Scientific, Pittsburgh, PA, USA) at 4°C. Dialysate is changed at least once to ensure removal of unreacted molecules.

Characterization of ANXA5-DM1

The ANXA5-DM1 bioconjugate was characterized by absorbance spectroscopy, SDS-PAGE gel electrophoresis, and denaturing mass analysis. In the spectroscopic assay, the concentration of DM1 in the ANXA5-DM1 conjugate was determined by measuring the absorbance at 288 nm (peak absorbance for DM1) and then subtracting the absorbance contributed by the protein at this wavelength and taking into account the protein concentration, which was determined by the Bradford protein assay. SDS-PAGE analysis was also used to validate drug loading. ANXA5-DM1 was denatured in 2x Laemelli sample buffer (Bio-Rad, Hercules, CA, USA) and heated to 100°C for 5 min. Samples were transferred to 4-20% mini-protean TGX precast 10-well gradient gels (Bio-Rad, Hercules, CA, USA). The samples were run at 200 V for 30 minutes in 1% tris-glycine-SDS running buffer (Bio-Rad, Hercules, CA, USA), stained with Imperial stain (Thermo Fisher, Waltham, MA, USA), and destained with DI water overnight.

For the denaturing intact mass analysis (performed at the Department of Chemistry and Molecular Biology, University of Texas Medical Branch, Galveston, TX), samples containing purified ANXA5-DM1 (0.14 mg/mL final concentration) were analyzed using size exclusion chromatography (SEC) coupled directly to mass spectrometry. Sample injection (1 µL) and on-line SEC-desalting was accomplished using an organic/acidic mobile phase (30% acetonitrile, 0.1% formic acid, 0.02% trifluoroacetic acid) pumped with a Vanquish Horizon UHPLC system (Thermo Scientific) through an SEC UHPLC column (BEH200 SEC 4.6 x 50 mm, Waters), at a flow rate of 20 µL/min. The LC flow path was connected inline with an Orbitrap Eclipse Tribrid mass spectrometer (Thermo Scientific) via a heated electrospray ionization (HESI-II) ion source (Thermo Scientific). The ion source was operated with 3500 V spray voltage, sheath gas setting of 40 units, auxiliary gas setting of 10 units, and vaporizer temperature of 75˚C. The MS capillary temperature was set to 325˚C. Intact mass spectra were generated by using an RF Lens setting of 50 V to desolvate protein ions, isolating a mass range of m/z 500-450, and reacting isolated ions with proton transfer charge reduction for 6 ms, followed by scanning for charge reduced intact protein product ions using a mass range of m/z 500 – 8000. Intact protein ions were detected in the Orbitrap, scanning at a resolution setting of 50,000 (at m/z 200). Raw LC-MS data were analyzed using BioPharma Finder (Thermo Scientific) software. LC-MS spectra representing intact ANXA5-DM1 profiles were deconvoluted using the ReSpect and Sliding Window algorithms with a ReSpect mass tolerance of 20 ppm, a Sliding Window mass tolerance of 20 ppm, and a deconvolution mass range of 35 – 45kDa [2]. Intact mass assignments of individual ANXA5-DM1(n) conjugate isoforms were based on tolerance of 100 ppm. The drug-to-protein ratio was calculated as a weighted average of the ReSpect-Sliding Window abundances for each of the ANXA5-DM1(n) isoforms detected.

Cell lines and culture conditions

All cell lines and cell media were purchased from American Type Culture Collection (ATCC, Manassas, VA, USA). EMT6 murine breast carcinoma cells were cultivated with Waymouth's MB 752/1 medium (Sigma-Aldrich, St. Louis, MO, USA) supplemented with 2 mM glutamine, 15% FBS, and 1% penicillin/streptomycin antibiotics. 4T1 murine breast cancer cells were cultivated with Roswell Park Memorial Institute 1640 medium (RPMI-1640) supplemented with 10% FBS and 1% penicillin/streptomycin. MCF10A nontumorigenic human breast epithelial cells were cultured in mammary epithelial growth medium (MEGM) with MEGM bullet kit (Lonza, Greenwood, SC, USA) (2 mL BPE, 0.5 ml hEGF, 0.5 ml insulin, 0.5 ml hydrocortisone, 0.5 ml GA); GA-1000 was omitted due to ATCC recommendations. Additionally, ATCC recommends 100 ng/ml cholera toxin (Sigma-Aldrich, St. Louis, MO, USA), which was added to the MCF10A cell culture medium as well as 1% antibiotic-antimycotic. All TNBC were passaged using 0.25 % (w/v) trypsin in 0.53 mM EDTA (Thermo Fisher Scientific, Waltham, MA, USA) and neutralizing trypsin with fully supplemented medium. MCF10A utilized the same trypsinization procedure but utilized soybean trypsin inhibitor for trypsin neutralization. All cell lines were cultured under a 5% CO_2_-supplemented atmosphere at a temperature of 37°C and 100% relative humidity. The medium was refreshed every 48 h. When plated for studies, adherent cancer cell cultures were grown to less than 75% confluence, and healthy cell lines cells were grown to 100% confluency to mimic the conditions in healthy tissue.

4.5 ANXA5 colorimetric binding

Binding strength was analyzed via a modified indirect ELISA [3, 4]. Cells (50,000 cells/well) were grown onto 24 well plates and fixed with 0.025% glutaraldehyde once cancer cells were 70-80% confluent, or MCF10A noncancerous cells were 100% confluent. Cancer cells were blocked with 0.5% bovine serum albumin (BSA) for 1 h at 37°C. Cells were incubated with 0-20 nM of biotinylated ANXA5 for 2 h at 37°C and 5% CO_2_. ANXA5 was biotinylated with a Roche biotin protein labeling kit (Roche, Basel, Switzerland), and the level of biotinylation was quantified with a Pierce Biotin Quantification kit (Pierce Biotechnology, Waltham, MA, USA) per manufacturer’s instructions. Cells were washed four times with 0.5% BSA to remove unbound ANXA5. Cells were then seeded with 2 μg/ml streptavidin-horseradish peroxidase (Strep-HRP) for 1 h at room temperature. Cells were washed four times with 0.5% BSA to remove unbound Strep-HRP, and the chromogenic substrate O-phenylenediamine (OPD) and 30% hydrogen peroxide were added to each well to induce a yellow color change. Cells were incubated at room temperature in the dark. The supernatant was collected, and the absorbance was read at 450 nm on a BioTek Synergy HT microtiter plate reader (Winooski, VT). For each concentration of biotinylated ANXA5, the specific binding was obtained by subtracting non-specific binding, when no calcium was present (biotinylated ANXA5 with 5 mM of EDTA), from the total binding (biotinylated ANXA5 with 2 mM CaCl_2_). The dissociation constant was determined using the nonlinear regression one-site total and nonspecific binding model in GraphPad Prism version 9 software (Graph Pad, San Diego California, USA).

ANXA5-DM1 and ANX5 cell viability assay

Cell viability was assayed by a resazurin assay (AlamarBlue assay, n=3-5). Cancer cells were seeded at 1,000 cells/well in 96 well plates and treated 24-48 h after seeding. Healthy cells were seeded at 10,000 cells per well in 96 well plates and treated once cells reached 100% confluency. For ANXA5-DM1 cytotoxicity studies, cells were then treated with 0-100 μM of DM1 in the ANXA5-DM1 conjugate or free DM1 for 72 h in fully supplemented growth medium supplemented with 2 mM calcium to promote ANXA5 binding. For ANXA5 cytotoxicity studies, cells were treated with 0-0.7 μM of ANXA5 for 72 h in fully supplemented growth medium supplemented with 2 mM calcium to promote ANXA5 binding. Following incubation with the drug, cell viability was assayed by resazurin dye reduction assay using AlamarBlue (Thermo Fisher Scientific, Waltham, MA, USA) as per manufacturer instructions and recorded using a Synergy HTX multi-mode microtiter plate reader (BioTek, Winooski, VT, USA). The concentration to inhibit cell growth by 50% (IC50) was derived from the dose-response curves by using the sum of squared differences to fit a sigmoidal regression of the form:

$$V=\frac{Max}{(1+\frac{C}{\text{IC}50})H}$$

where *V* is the response (viability), *Max* is the theoretical maximum response (100% viability), *C* is the concentration of the drug, and *H* is the Hill coefficient that describes how “steep” the curve is.

Immunogenic cell death - ATP release and calreticulin externalization

To determine if ANXA5-DM1 induces ATP release from cells after treatment, an ATP luminescence kit (Molecular Probes, Eugene, OR, USA) was utilized according to the manufacturer’s instructions. Briefly, EMT6 and 4T1 cells were seeded at 5,000 cells/well in 96 well plates and treated 24 h after seeding. Cells were treated with 0 or 10 nM of DM1 in the ANXA5-DM1 conjugate or free DM1 for 24 h. After 24 h, 10 μL of cell media was added to 90 μL of standard reaction solution in a white plate. Luminescence reading was obtained from the plate reader, and ATP concentration was calculated by comparing the luminescence reading to a standard curve.

To determine if ANXA5-DM1 induces calreticulin surface expression, flow cytometry was utilized. EMT6 and 4T1 cells were seeded at 25,000 cells/well in 24 well plates and treated 24 h after seeding. Cells were treated with 0 or 10 nM of DM1 in the ANXA5-DM1 conjugate or free DM1 for 24 h. After 24 h, media was collected into microcentrifuge tubes to collect dead cells. To ensure there would be enough cells, three wells were combined to be one sample. Cells in the plates were then washed with PBS, and 100 μL of trypsin was added to each well. Once 90% of the cells were removed from the plate (2-5 min), cells were added to the microcentrifuge tubes and centrifuged at 500 x g for 5 min. Cells were resuspended in 200 μL of 0.5% BSA-PBS and counted to have at least 100,000 cells per sample. Cells were then fixed with 4% paraformaldehyde (PFA, Thermo Fisher Scientific, Waltham MA, USA) for 20 min at 4°C. Cells were then washed two times with 500 μl of 0.5% BSA-PBS at 500 x g for 5 minutes. After the second wash, cells were resuspended in 100 μl of 0.5% BSA-PBS, and 1 μl of CD16/CD32 Fc Block (eBioscience, San Diego, CA, USA) was added. Cells were then incubated for 20 min at 4°C. Finally, 1 μl of FITC-labeled calreticulin (Novus Biologicals, Centennial, CO, USA) was added. Cells were incubated for another 30 minutes at 4°C in the dark. After incubation, cells were analyzed on a BD Biosciences Accuri C6 flow cytometer (Franklin Lakes, New Jersey, USA) with excitation at 488 nm, and a 533/30 bandpass filter was used to capture 10,000 gated events per sample. Data was collected and analyzed with BD C6 Accuri software. One sample was composed of three wells, and each sample was analyzed three times. All experiments were conducted in triplicate.

Statistics

All statistical analysis was performed with GraphPad Prism statistical suite version 8 or 9. A student’s T-test was performed for dose-specific cytotoxicity studies that utilized two groups. A one-way analysis of variance (ANOVA) was performed for ANXA5 cytotoxicity, ATP release, and calreticulin surface expression that used three or more groups. Tukey-Kramer multiple comparison post hoc analysis was utilized to determine the statistical significance between the groups. A cutoff value of p < 0.05 indicated significance for all studies.

**Results**

Characterization of ANX5-DM1 by absorbance

To determine the average number of DM1 molecules per ANXA5 protein, the absorbance of a sample of the conjugate and a sample of the same protein concentration of unconjugated annexin was measured at 288 nm (DM1 peak absorbance). The peaks were subtracted from each other to find the contribution of only DM1 to the absorbance at 288 nm. The resulting absorbance value was compared to a standard curve of DM1 concentrations in solution to determine the concentration of DM1 on the proteins. The molar concentration of DM1 was divided by the molar concentration of the AV protein to arrive at the average DM1 per AV loading.

A loading of 6:1 molecules of DM1 to one ANXA5 molecule was determined. The result of the absorbance versus wavelength after subtraction of the free ANXA5 absorbance is shown in Supplemental Fig. 1.


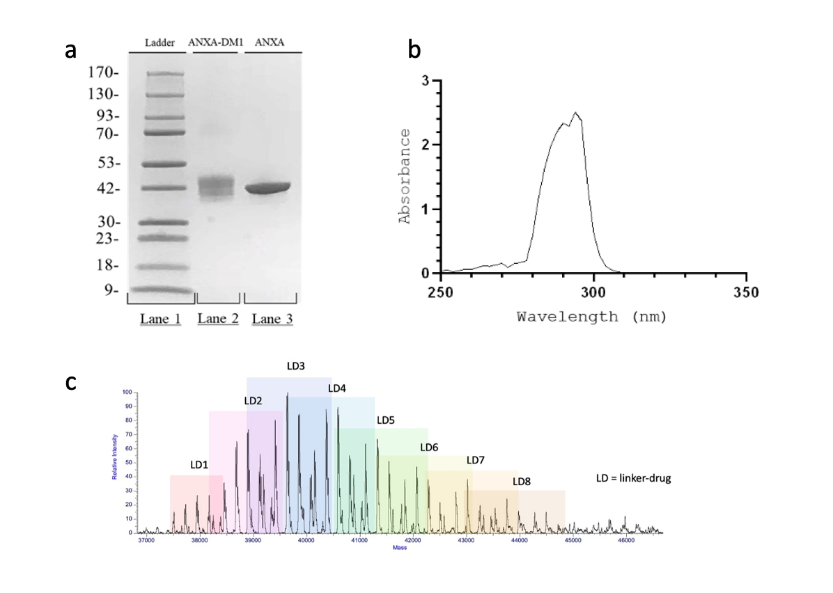


**Supplemental Fig. 1**: Characterization of ANXA5-DM1 by absorbance at 288 nm. Absorbance spectroscopy (OD: 288 nm) of DM1 following spectral correction for ANXA5 for detection of DM1.

Binding of ANXA5 to breast cancer cells and healthy breast cells

To confirm the ability of the ANXA5 to bind to PS on the surface of TNBC cells, equilibrium binding experiments with increasing concentrations of biotinylated ANXA5 were used. Cells were treated with 0-20 nM of ANXA5, and total, non-specific, and specific binding were obtained. Total binding was obtained by supplementing 0.5% BSA with 2 mM Ca^2+^ to promote ANXA5 binding. Non-specific binding was obtained by supplementing 0.5% BSA with 5 mM EDTA, a calcium-chelating agent. The EDTA removes excess calcium and inhibits ANXA5 binding. Specific binding was obtained by subtracting non-specific binding from total binding. After subtracting out 0 nM ANXA5 background absorbance, the non-specific binding was essentially 0, meaning ANXA5 was not binding to the EMT6 and 4T1 cells when Ca^2+^ was absent. The total and specific binding were nearly identical indicating the binding of ANXA5 to PS is calcium-dependent (Supplemental Fig. 2).

The dissociation constant for ANXA5 binding to 4T1 and EMT6 cells was found to be in the low nanomolar range, indicating strong binding (Supplemental Table 1). A dissociation constant for healthy MCF10A human breast cells grown to confluence could not be determined because the total binding and the nonspecific binding overlapped, indicating no specific binding to the cells was observed.

**Supplemental Table 1:** ANXA5 dissociation constants on 4T1, EMT6, and MCF10A cell lines

| **Cell line** | **Dissociation constant (K_d_)** |
| --- | --- |
| EMT6 | 1.14 nM |
| 4T1 | 2.31 nM |
| MCF10A | Not detectable |


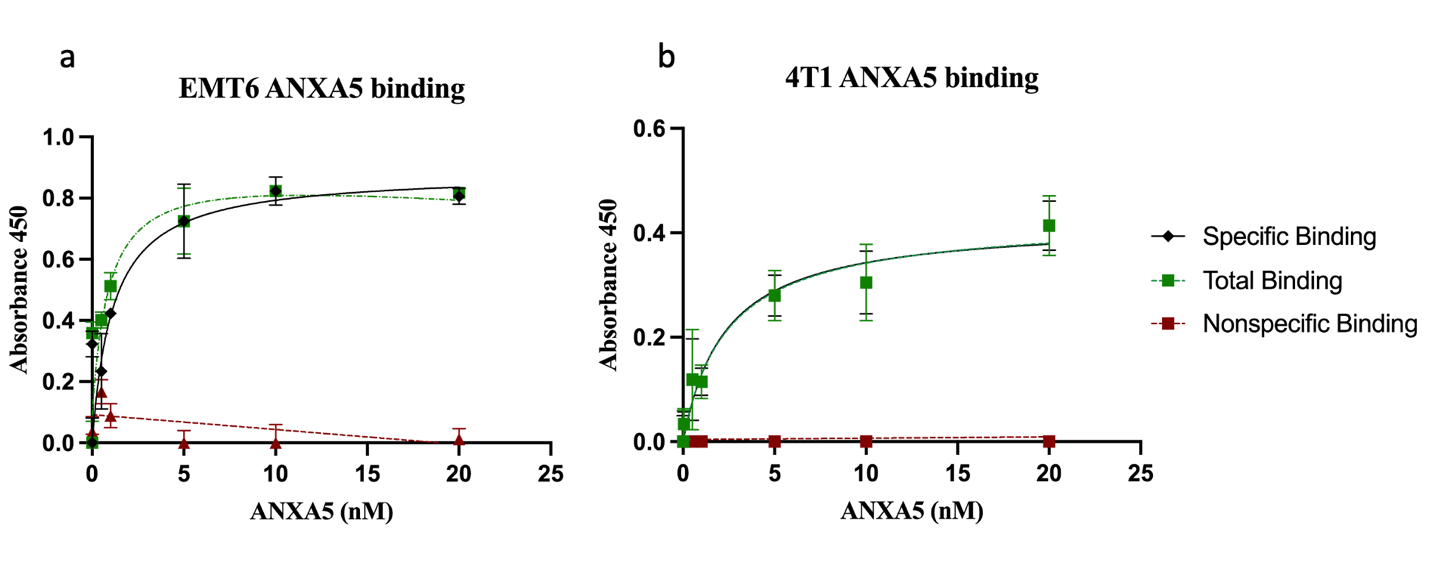


**Supplemental Fig. 2**: Binding strength of ANXA5 on TNBC with PS externalization

**a** EMT6 and **b** 4T1 cells were incubated with 0-20 nM of ANXA5. Total binding (green square with dot-dash line) was measured with the addition of 2 mM Ca^2+^ to promote ANXA5 binding. Nonspecific binding (red triangle with dashed line) was measured with the addition of EDTA to chelate residual Ca^2+^, inhibiting ANXA5 binding. Specific binding (black square with solid line) was obtained by subtracting nonspecific binding from total binding. The dissociation constant of the specific binding was 1.14 nM for EMT6 cells and 2.31 nM for 4T1 cells. Data presented as mean ± SD (n = 3).

**References**

1. Neves LF, Van Rite BD, Krais JJ, Ramesh R, Resasco DE, Harrison RG. Targeting single-walled carbon nanotubes for the treatment of breast cancer using photothermal therapy. Nanotechnology. 2013;24:375104.

2. Bailey AO, Han G, Phung W, Gazis P, Sutton J, Josephs JL et al. Charge variant native mass spectrometry benefits mass precision and dynamic range of monoclonal antibody intact mass analysis. MAbs. 2018;10:1214-25. doi:10.1080/19420862.2018.1521131.

3. Krais JJ, de Crescenzo O, Harrison RG. Purine Nucleoside Phosphorylase Targeted by Annexin V to Breast Cancer Vasculature for Enzyme Prodrug Therapy. PLoS ONE. 2013;8:e76403. doi:10.1371/journal.pone.0076403.

4. Van Rite BD, Harrison RG. Annexin V-targeted enzyme prodrug therapy using cytosine deaminase in combination with 5-fluorocytosine. Cancer Letters. 2011;307:53-61. doi:10.1016/j.canlet.2011.03.016.
